# Information Guided Exploration of Scalar Values and Isocontours in Ensemble Datasets

**DOI:** 10.3390/e20070540

**Published:** 2018-07-20

**Authors:** Subhashis Hazarika, Ayan Biswas, Soumya Dutta, Han-Wei Shen

**Affiliations:** 1 GRAVITY Research Group, Department of Computer Science and Engineering, The Ohio State University, Columbus, OH 43218-2646, USA; 2 Los Alamos National Laboratory, Los Alamos, NM 87545, USA

**Keywords:** specific information, ensemble visualization, uncertainty visualization

## Abstract

Uncertainty of scalar values in an ensemble dataset is often represented by the collection of their corresponding isocontours. Various techniques such as contour-boxplot, contour variability plot, glyphs and probabilistic marching-cubes have been proposed to analyze and visualize ensemble isocontours. All these techniques assume that a scalar value of interest is already known to the user. Not much work has been done in guiding users to select the scalar values for such uncertainty analysis. Moreover, analyzing and visualizing a large collection of ensemble isocontours for a selected scalar value has its own challenges. Interpreting the visualizations of such large collections of isocontours is also a difficult task. In this work, we propose a new information-theoretic approach towards addressing these issues. Using specific information measures that estimate the predictability and surprise of specific scalar values, we evaluate the overall uncertainty associated with all the scalar values in an ensemble system. This helps the scientist to understand the effects of uncertainty on different data features. To understand in finer details the contribution of individual members towards the uncertainty of the ensemble isocontours of a selected scalar value, we propose a conditional entropy based algorithm to quantify the individual contributions. This can help simplify analysis and visualization for systems with more members by identifying the members contributing the most towards overall uncertainty. We demonstrate the efficacy of our method by applying it on real-world datasets from material sciences, weather forecasting and ocean simulation experiments.

## 1. Introduction

Ensemble datasets are one of the primary sources of uncertain datasets in scientific studies. While modeling and measuring a real world phenomenon via simulations, the lack of knowledge regarding the ground truth compels scientists to use multiple initial conditions and/or different input parameters to get an estimate of the possible outcomes. The resulting ensemble datasets are used for decision making in real world and thus, are of prime importance to the weather and geo-scientists. This has led researchers to focus on the specific field of uncertainty analysis over the past few years [1,2,3].

For an effective exploration of scalar ensemble datasets, use of uncertain isocontours has been a popular method and has attracted significant attention recently. Besides the popular spaghetti plot, various techniques such as contour-boxplot [4], circular glyphs [5], contour variability-plot [6], and probabilistic marching-cubes [7] have been proposed to visualize ensemble isocontours. All these existing works conduct uncertainty analysis by assuming that a particular scalar value of interest is already known. Since not all scalar values have the same degree of isocontour uncertainty, scientists trying to study the effects of multiple simulations/runs on a range of scalar values may lack a thorough understanding of the uncertainty associated with all the values. Such an analysis of uncertainty across all the scalar values can help scientists as well as visualization practitioners to identify interesting scalar values for further analysis using some of the aforementioned isocontour analysis techniques. Note that, for a single selected scalar value, not all the individual members contribute equally to the overall uncertainty of the ensemble isocontour structure. The existing ensemble isocontour analysis techniques do not offer insights into the contribution of individual members towards the uncertainty. Understanding the contribution of individual members is essential not only to comprehend the important simulations/runs from a large collection of members but also to understand the effect of uncertainty on the scalar value in the experiment. To date, a single coherent analysis framework that analyzes the uncertainty of both the scalar value range as well as the ensemble isocontours of an individual scalar value is mostly missing. Our work is an effort to fill this gap and address the above unaddressed facets of uncertainty analysis in ensemble datasets.

In this paper, we introduce a two-stage information-theoretic framework for the exploration of the scalar values as well as their corresponding ensemble isocontours for ensemble scalar datasets. In the first stage, to understand the effect of uncertainty on all the scalar values and eventually guide the user towards selecting interesting scalar values for further analysis, we evaluate the ensemble isocontour variations across all the scalar values. Using two specific information measures, we compute the predictability and surprise of specific scalar values. Predictability of a scalar value conveys the relative similarity of the corresponding ensemble isocontours, while surprise conveys the relative importance of the scalar values in the field. Surprise along with predictability help us quantify the importance as well as the uncertainty of the scalar values in ensemble datasets. Therefore, the non-trivial problem of evaluating the uncertainty of the ensemble isocontours for all the individual scalar values can be efficiently addressed by our proposed analysis method. To facilitate the identification and selection of interesting scalar values for further exploration, we present an interactive scatter plot view which is linked to a violin plot [8] view showing the distribution of individual predictability values of the members. For values with a high variation in their predictability, it is worth investigating the cause of uncertainty and identifying the contribution of individual member isocontours towards the overall uncertainty. This leads to the next stage of our information guided exploration of the ensemble isocontours of a single scalar value.

We propose a conditional entropy based algorithm to identify the contribution of individual members towards the overall uncertainty of the ensemble isocontours for a single scalar value. Since individual members contribute differently toward the total structural uncertainty of an ensemble of isocontours, use of conditional entropy provides an effective measure to identify the relative importance of the members by accounting for the information overlap among their corresponding isocontours. To assist the users with exploration of the relative importance of individual isocontours for a selected scalar value, we provide an interactive information gain curve. This curve conveys the overall information gained about the complete set of ensemble isocontours by selecting a specific sequence of members. Besides exploring the relative importance of each isocontour, it also helps us to select representative subsets/samples of isocontours from large number of members that can represent the uncertainty of all the members.

To summarize, the major contributions of our work are twofold:

- Using specific information measures, we evaluate the ensemble isocontour uncertainty of all the scalar values in an efficient and effective way.
- For a single scalar value, we propose a conditional entropy based approach to identify the contributions of individual members towards the uncertainty of the ensemble isocontours.

## 2. Related Work

### 2.1. Information Theory in Visualization

Many visualization and computer graphics problems have been solved using information theory [9]. The recently published book, Information Theory in Visualization [10], covers in great details how information theory helped solve many challenging problems in visualization. For time-varying datasets, Wang et al. [11] performed an information based block-wise analysis to identify important time varying features. Chen and Janicke [12] provided evidence that information theory can be used to analyze many visualization problems. For flow visualization, an information theoretic framework was provide by Xu et al. [13] to evaluate the effectiveness of visualizations in communicating the original data information to the users. One popular information-theoretic measure is mutual information. Mutual information has been used in the medical image registration and multi-modal data analysis for a long time now [14,15]. Bruckner et al. [16] used mutual information to measure the similarity of isosurfaces in scientific datasets and proposed the isosurfaces similarity map to identify salient isosurfaces. Wei et al. [17] used similar mutual information based method to evaluate isosurfaces for surface morphing. Specific information measures are essentially decompositions of mutual information. $I_{1}$ and $I_{2}$ specific information measures were first introduced in the works of DeWeese and Meister [18]. Bramon et al. [19] used them to perform fusion of multi-modal images. Dutta et al. [20] used mutual information and its various decompositions to select important isosurfaces in multivariate time-varying datasets. Biswas et al. [21] proposed an information-theoretic framework for multivariate data analysis. They used mutual information, specific information and conditional entropy to identify salient isocontours in multivariate datasets. In the field of pattern recognition and feature selection, various mutual information based tools and techniques have been proposed to optimally select features based on criterion such as minimizing redundancy and maximizing relevance [22,23]. However, techniques for feature analysis and selections which meet the needs of ensemble scientific data is mostly missing. In our work, we propose an information theoretic approach towards understanding the effect of uncertainty in scalar values and their features (isocontours) in ensemble datasets.

### 2.2. Ensemble Data Visualization

Visualization of ensemble data falls in the general category of uncertainty visualization. The field of uncertainty visualization has seen many innovations over the past two decades [2,3,24,25]. Potter et al. [26] provided an extensive survey of the sources of uncertainty in data as well as possible visualization based answers for analyzing them in different dimensions. Ensemble dataset is a special category of uncertain datasets where data are generated from multiple simulations or runs with varying parameter settings. This type of dataset is very popular in the field of weather forecasting and simulation sciences [27,28]. Obermair et al. [29] categorized the different ensemble visualization techniques based on their approaches and discussed the possible future challenges in ensemble visualization. Wang et al. [30], in a recent survey of visualization and visual analysis techniques for ensemble data, provided a structured view of the general approaches of dealing with ensemble data analysis. Potter et al. [31] built a comprehensive framework called Ensemble-Vis to visualize 2D weather forecasting and climate modeling ensembles using multiple statistical visualization techniques. Demir et al. [32] tried addressing the challenges of 3D ensemble data by using multi-chart visualizations. For ensemble datasets, effective isocontour visualization is a very challenging task. In meteorology, spaghetti plots are commonly used to display simultaneously all the ensemble isocontours. Sanyal et al. [5] introduced a tool called Noodles which enhanced spaghetti plots by using circular glyphs and confidence ribbons to highlight the spread of isocontour lines. Alabi et al. [33] proposed Ensemble Surface Slicing (ESS) to show the variation of an ensemble of isosurfaces. Hazarika et al. [34] visualized the order-statistics of ensemble isosurfaces of multiple isovalues using parallel-coordinate systems. Attempts have been made to create uncertain isocontours using the probabilities of level-set crossing [35,36,37]. This led to the concept of probabilistic marching cubes by Pothkow et al. [7], used to extract uncertain isosurfaces from a distribution field. To address the quantitative aspect of ensemble isocontour visualizations, Whitaker et al. [4] proposed contour boxplot to visualize the statistical properties, outliers and other variabilities of contours. Recently, Ferstl et al. [6] proposed a contour variability plot by clustering groups of ensemble isocontours. In all these uncertainty analysis techniques, it is assumed that a certain scalar value of interest is already known to the users. To the best of our knowledge, not much work has been done to help the users in selecting such scalar values for uncertain isocontour analysis. The method proposed in this paper gives the users an overview of the uncertainty of different scalar values to let them select specific isovalues for further analysis.

## 3. System Overview

**Figure 1:**
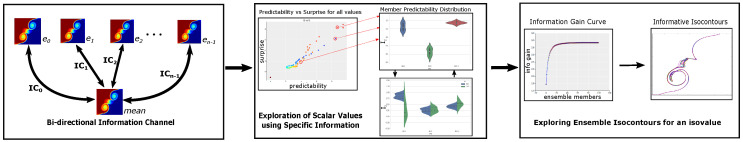
A schematic overview showing the main stages of our proposed information-theoretic method.

In this section, we provide a brief overview of the proposed information guided exploration of scalar values and their isocontours in ensemble datasets. Figure 1 gives a schematic overview of our method. To explore the uncertainty associated with ensemble isocontours of all the scalar values, we use specific information measures which evaluate the predictability and surprise of specific scalar values. We first establish bi-directional information channels, $IC_{i}$, between the scalar fields of individual members *i* and the mean field as illustrated in Figure 1. In the absence of a ground-truth, mean field is a popular and widely accepted single field representation of the members [3,31,38]. By computing the specific information measures of each member against the mean reference field, we can compute the total predictability and total surprise of the scalar values. Scalar values with high total predictability indicate low uncertainty of their corresponding ensemble isocontours, whereas low predictability indicates high variation in their ensemble isocontours, i.e., high uncertainty. On the other hand, surprise is an indicator of the importance of the feature based on the frequency of the corresponding scalar values. We convey this information in the form of an interactive predictability versus surprise scatter plot view. This is complemented with two linked violin plot views which visualize the distributions of individual predictability values for the user selected scalar values in the scatter plot. For scalar values showing high deviation of its individual predictability values, it is important to understand the contribution of individual members to the overall uncertainty of that value. In the second stage of our exploration, we investigate the contribution of each member to the overall uncertainty of the ensemble isocontours of a single scalar value. We propose a conditional entropy based isocontour selection algorithm to identify the most informative isocontours. To assist the users in selecting the informative isocontours, we create an interactive information gain curve that conveys the information gained by selecting a particular set of members.

## 4. Method

Below, we discuss in detail the two main stages of our proposed information guided exploration process. We first explain the process of scalar value exploration, followed by the exploration of informative isocontours of a selected isovalue.

### 4.1. Specific Information Based Scalar Value Exploration

The ensemble isocontours corresponding to different scalar values show different degrees of structural variations. This is because all the scalar values are not equally affected by uncertainty in ensemble simulation experiments. Exhaustively extracting the individual ensemble isocontours for all the scalar values and analyzing their uncertainty is computationally prohibitive for large number of ensemble members. Instead of extracting the isocontours individually, we use information-theoretic measures called specific information, which allow us to evaluate the structural variations and, hence, the uncertainty of the ensemble isocontours. Specific information measures are essentially decompositions of mutual information that let us evaluate the information content of specific instances/realizations of a random variable. As a result, by considering the scalar fields as random variables, we can efficiently analyze multiple scalar values using specific information measures. In particular, we use $I_{1}$ and $I_{2}$ specific information measures [18,19], which evaluate the surprise and predictability of specific scalar values, respectively. Predictability offers a measure of how similar the corresponding ensemble isocontours of a specific scalar value are, while surprise corresponds to the relative importance of the scalar value in the field based on their frequency. Instead of computing these measures for all pairs of ensemble members, which is an exponentially expensive task for large ensemble systems, we evaluate them for each member against the mean scalar field. For a system with *n* ensemble members, $\left\{e_{1},e_{2},\ldots e_{n}\right\}$, the value at a grid location (x,y), in the mean scalar field can be denoted as $\frac{1}{n}\sum_{i=1}^{n}e_{i}\left(x,y\right)$. The mean scalar field acts as a frame of reference for comparing the individual specific information measures of the members. This also allows us to quantify how well the scalar values are represented by the mean scalar fields, which is often utilized by scientists to aggregate or summarize ensemble results into a single field [3,31]. To compute these values, we first establish bi-directional information channels between each of the member scalar fields and the mean field.

#### 4.1.1. Information Channels

Information channel between two scalar fields can be denoted as *X*→*Y*, where *X* and *Y* are the input and output random variables representing the two scalar fields. The three basic components of the channel *X*→*Y* are:

- Input distribution p(X) represents the normalized frequency of each scalar value *x* in the distribution of *X*.
- Conditional probability distribution p(Y|X) expresses how the distribution of each of the scalar values (i.e., *x*) of the input field *X* match with the distribution of output field *Y*.
- Output distribution p(Y) represents the normalized frequency of each scalar value *y* in the distribution of *Y*.

Throughout this paper, we use *x* to refer to a single scalar value in the scalar value distribution of the field corresponding to *X* (i.e., the bin center in the corresponding histogram).

In our work, we have used bi-directional information channels between the member fields and the mean as illustrated in the first stage in Figure 1. The direction “mean→member”, where input is the mean field and the output is a member field, helps us quantify how much information the member scalar fields retain about the scalar values of the mean field. In other words, it allows us to quantify how well the spatial distribution of the scalar values in the mean field represent the corresponding distribution in the individual member fields. On the other hand, the reverse direction, i.e., “member→mean”, lets us evaluate how much information the mean field possess about the scalar values in the individual member fields. Before we describe in detail how to compute the specific information measures of a channel, we briefly introduce two closely related information theory concepts i.e., entropy and mutual information.

#### 4.1.2. Entropy

Entropy provides a measure of the uncertainty associated with a random variable. If p(x) is the probability of event *x* (∼X), then the uncertainty associated with *X* can be described by Shannon’s entropy as:

(1) $$H\left(X\right)=-\sum_{x\in X}p\left(x\right)\log p\left(x\right)$$

Similarly, for two random variables *X* and *Y*, the joint entropy can be described as:

(2) $$H\left(X,Y\right)=-\sum_{y\in Y}\sum_{x\in X}p\left(x,y\right)\log p\left(x,y\right)$$

#### 4.1.3. Mutual Information

Mutual information is a measure of the information overlap between two random variables. For the two random variables *X* and *Y*, mutual information can be denoted as:

(3) $$I\left(X,Y\right)=\sum_{y\in Y}\sum_{x\in X}p\left(x,y\right)\log\frac{p(x,y)}{p(x)p(y)}$$

Mutual information between two random variables can also be described as the amount of uncertainty reduced about one variable after observing the other variable. In other words, after observing the variable *X*, the amount of uncertainty reduced about variable *Y* can be denoted as:

(4) I(X,Y)=H(Y)−H(Y|X)

#### 4.1.4. Specific Information

In our work, we use two types of specific-information measures, namely, $I_{1}$ and $I_{2}$ [18], which are essentially two different forms of decomposition of the popular mutual information measure. They were also referred to as surprise and predictability by Bramon et al. [19] based on the type of information that they quantify. We apply these measures to propose an efficient analysis strategy for evaluating the effect of uncertainty across multiple scalar values in ensemble datasets.

#### 4.1.5. Surprise ($I_{1}$)

Surprise or $I_{1}$ of *x* (instance of input distribution *X*) with respect to the output distribution *Y* is given as:

(5) $$I_{1}\left(x;Y\right)=\sum_{y\in Y}p\left(y|x\right)\log\frac{p(y|x)}{p(y)}$$

$I_{1}\left(x;Y\right)$ essentially represents the Kullback–Leibler divergence [39] between p(Y|x) and p(Y). A high $I_{1}\left(x;Y\right)$ indicates that given the observed value *x* in *X*, certain low frequency occurrences y∈Y have become more probable, which account for an unlikely or surprising behavior. $I_{1}$ is an effective tool to understand the importance of a feature corresponding to a scalar value [21] based on the frequency of the value (i.e., the size of the feature). In general, for scientific datasets, low frequency scalar values correspond to interesting foreground features (high surprise), while very high frequency values often correspond to background features (low surprise). Therefore, $I_{1}$ or surprise helps to distinguish such important scalar values across the ensemble members in scientific datasets.

#### 4.1.6. Predictability($I_{2}$)

Predictability or $I_{2}$ is based on the change of entropy of the channel and is given as:

(6) $$\begin{array}{cc} I_{2}\left(x;Y\right) & =H(Y)-H(Y|x)\\ & =-\sum_{y\in Y}p\left(y\right)\log p\left(y\right)+\sum_{y\in Y}p\left(y|x\right)\log p\left(y|x\right) \end{array}$$

$I_{2}\left(x;Y\right)$ gives the amount of reduction in uncertainty about *Y* after observing the data value *x*. Generally, a high $I_{2}\left(x;Y\right)$ indicates that given the observed value *x* in *X*, we can predict the corresponding *y*s in *Y* with high confidence. Therefore, high predictability corresponds to less uncertainty of the scalar values and vice versa.

#### 4.1.7. Relationship with Mutual Information

Both the specific information measures are different decompositions of the mutual information measure. Therefore, the average information gained from $I_{1}$ and $I_{2}$ for all the specific realizations of a variable is equal to the overall mutual information. For $I_{1}$, using Equation (3), this relationship can be expressed as:

(7) $$\begin{array}{cc} \sum_{x\in X}p\left(x\right)I_{1}\left(x;Y\right) & =\sum_{x\in X}\sum_{y\in Y}p\left(x\right)p\left(y|x\right)\log\frac{p(y|x)}{p(y)}\\ & =\sum_{x\in X}\sum_{y\in Y}p\left(x,y\right)\log\frac{p(x,y)}{p(x)p(y)}\\ & =I(X,Y) \end{array}$$

Further, for $I_{2}$, using Equation (4), this relationship can be expressed as:

(8) $$\begin{array}{cc} \sum_{x\in X}p\left(x\right)I_{2}\left(x;Y\right) & =\sum_{x\in X}p\left(x\right)H\left(Y\right)-\sum_{x\in X}p\left(x\right)H\left(Y|x\right)\\ & =H(Y)-H(Y|X)\\ & =I(X,Y) \end{array}$$

#### 4.1.8. Synthetic Data

**Figure 2:**
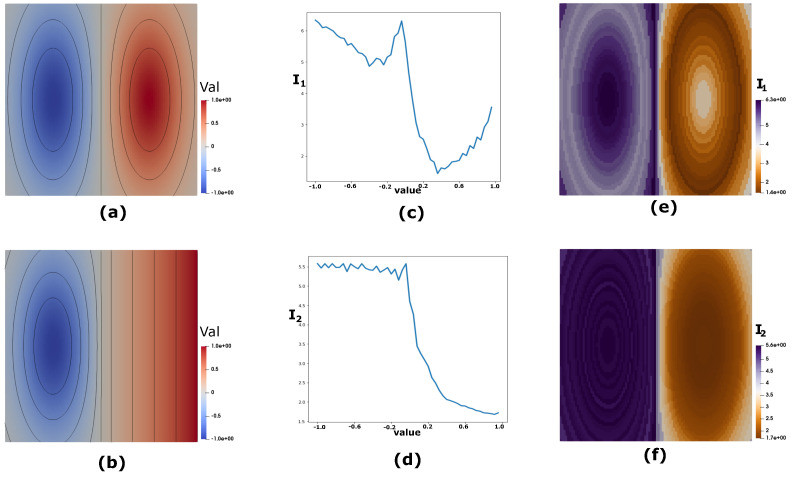
Synthetic Data: (**a**) Scalar field with two Gaussian structures; (**b**) scalar field with one Gaussian structure in a linearly increasing field; (**c**) the $I_{1}$ plot for the scalar values of (**a**) with respect to the field in (**b**); (**d**) the $I_{2}$ plot for the scalar values of (**a**) with respect to the field in (**b**); (**e**) the $I_{1}$ values color-mapped to the scalar values of the field (**a**); and (**f**) the $I_{2}$ values color-mapped to the scalar values of the field (**a**).

Figure 2 highlights the utility of these two measures in evaluating the uncertainty of scalar values using two synthetic datasets. Consider two synthetic datasets as shown in Figure 2a,b. Let random variables *X* and *Y* represent the two scalar fields shown in Figure 2a,b, respectively. *X* is created by mixing two Gaussian fields, while *Y* consists of a single Gaussian added to a linearly increasing field. The highlighted isocontours in black, as shown in Figure 2a,b reveals the underlying scalar field structure. Consider an information channel *X*→*Y*, which can be used to calculate the specific information measures $I_{1}$ (surprise) and $I_{2}$ (predictability) of specific scalar values x∈X. The results of these two measures for all *x*s in *X* is shown by the plots in Figure 2c,d, respectively. Figure 2e,f shows the *X* field color-mapped to their $I_{1}$ and $I_{2}$ values of the scalar values, respectively, which provide a spatial perspective to the surprise and predictability of the field *X*. As can be seen in Figure 2d,f, the $I_{2}$ (predictability) values for the low scalar values corresponding to the left half of *X* have high predictability about the corresponding scalar values in *Y*. However, this predictability sharply decreases for the higher values which corresponds to the right halves of *X* and *Y*. In addition, within this high value range, predictability drops gradually for the higher scalar values. This is because as the radius of the elliptic isocontours on the right half of *X* decreases (i.e., for high scalar values), the predictability of these values with respect to the corresponding vertical contours in the right half of *Y* decreases at the same time.

With respect to $I_{1}$ or surprise, which is an indicator of the importance of features based on the frequency of scalar values, we see a different trend. As shown in Figure 2c,e, the surprise is usually high for the low frequency values and low for the high frequency values. This trend is clearly visible in the left and the right halves of *X*. The high degree of feature alignment on the left half results in higher overall surprise in the left half than the right half of *X*. However, within these two halves, we observe the trend of low surprise for high frequency values and vice versa. These results also corroborate the fact that $I_{1}$ is more sensitive to the feature size as compared to $I_{2}$. Therefore, $I_{1}$ and $I_{2}$ together help us in profiling the uncertainty of all the scalar values in a field without having to extract the corresponding isocontours and analyzing them separately. Next, we show the utility of these measures in exploring the uncertainty of scalar values in ensemble scientific datasets.

#### 4.1.9. Exploration of Scalar Values

In our work, we compute the surprise and predictability measures for all the information channels as illustrated in Figure 1. The $I_{1}$ and $I_{2}$ values of individual channels quantify the information content between the mean reference field and the corresponding member field. For each scalar value, aggregated values of the two measures are obtained by summing the individual $I_{1}$ and $I_{2}$ values across all the members, thus representing the total surprise and total predictability respectively. As the first step towards an effective exploration of the scalar value range, we present an interactive scatter plot view of the total predictability versus total surprise of the scalar values. This plot depicts the overall variation or uncertainty of the ensemble isocontours of all the scalar values. The values with high total predictability ($I_{2}$) and high total surprise ($I_{1}$) refer to low uncertainty of their corresponding isocontours, while, a low predictability score highlights high variation among the ensemble isocontours for that value. On the other hand, surprise determines the importance of the scalar value in the field. Therefore, our scatter plot view reveals the uncertainty as well as the relative importance of the scalar values in the studied ensemble field.

However, the scatter plot only conveys the overall uncertainty of the scalar values, it does not convey how the individual ensemble members are contributing to the uncertainty. To understand how the individual predictability values of the members are distributed, we show a secondary visualization in the form of violin plots [8] for the user-selected scalar values in the scatter plot. Violin plots can visualize both the order statistics (similar to a traditional box-plot) as well as the shape of the distribution of values. Therefore, we use it to reveal the distribution of individual predictability values. The shape of the violin glyph gives us a picture of how agreeing are the individual predictability scores of the members. We offer an additional split-view of the violin plot, where we visualize the distribution of individual predictability measures for both the directions of the bi-directional channels separately. The left side corresponds to the predictability of the values in the mean field (i.e., “mean→member”), while the right side shows the predictability distribution of the member fields (i.e., “member→mean”).

For values with high total predictability and low variations, it can be concluded that the mean field is a reliable representation and from an information-theoretic perspective, retains good amount of information about the individual members. However, for values showing low total predictability and high variation of individual predictability it is important to understand how the individual member isocontours contribute to the high uncertainty in detail. This leads us to the second stage of our information guided exploration, where we investigate the contribution of individual isocontours towards the overall uncertainty for a scalar value.

### 4.2. Conditional Entropy Based Isocontour Exploration

In the second stage of our exploration process, we provide the users with information-based guidance in analyzing the isocontours of a selected isovalue. Analysis and visualization of a large number of isocontours is not a trivial task. The members in an ensemble of isocontours are often of varying shapes. The overall variation of shape of all the members gives an idea of the uncertainty of that isovalue. However, not all isocontours contribute equally to the overall variations of the ensemble isocontours. We propose a novel conditional entropy based contour exploration technique which will not only provide a quantitative measure for the contribution of individual members to the overall uncertainty of the ensemble isocontours but will also help the users in selecting an informative sample/subset of contours from a large number of varying members.

**Figure 3:**
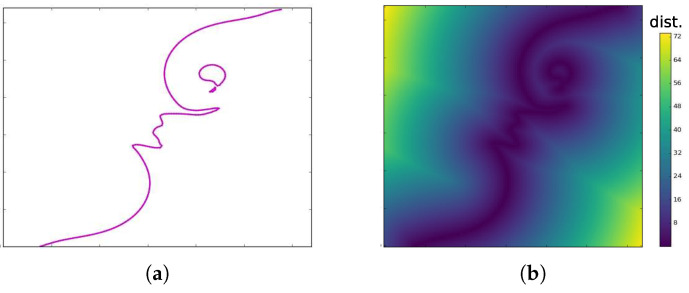
(**a**) Example isocontour; and (**b**) corresponding distance field transformation.

To apply information-theoretic measures on isocontours, they need to be first transformed into random variables that capture their respective shape information. This can be achieved by deriving an implicit representation of the isocontours. One popular implicit representation of isocontours is the distance field transformation [15]. The distance field is a spatial representation of a geometric object where at each point in the field we store the distance of that point to the closest point on the object [40]. If $C_{\theta }$ is the isocontour for isovalue θ then the Euclidean distance field transformation at a location *p* in the field is given as: $D_{\theta }\left(p\right)=\min_{\forall q\in C_{\theta }}dist\left(p,q\right)$. Figure 3 shows an isocontour and its corresponding distance field transformation. This implicit field for an isocontour can be considered as a random variable. Henceforth, by entropy of an isocontour, we mean the entropy of its corresponding distance field transformation.

#### 4.2.1. Conditional Entropy

Since our goal in this section is to select structurally informative isocontours with maximum contribution to the uncertainty of the system, choosing these contours based solely on their individual uncertainty or entropy does not suffice. Such entropy-based selection does not take into account the information overlap among the other existing contours of the system. For example, if there are two structurally similar contours that have high individual entropies, choosing just one of them will suffice in the current context because of their high information overlap. When one of these two contours is selected, the uncertainty remaining about the other will diminish significantly. In information theory, conditional entropy provides a method to select informative variables from a system of variables by taking into account the information overlap among all the system variables. For *n* given variables, $X_{1},\ldots ,X_{n}$, if *k* variables $X_{i1},\ldots ,X_{ik}$ are known then the amount of uncertainty left in the system is given by the following conditional entropy formula:

(9) $$H\left(X_{1},\ldots ,X_{n}|X_{i1},\ldots ,X_{ik}\right)=H\left(X_{1},\ldots ,X_{n}\right)-H\left(X_{i1},\ldots ,X_{ik}\right)$$

Here, $H(X_{1},\ldots ,X_{n})$ represents the joint entropy of the set of *n* variables $X_{1},\ldots ,X_{n}$ and is computed as:

(10) $$H\left(X_{1},\ldots ,X_{n}\right)=-\sum_{x_{1}\in X_{1}}\ldots \sum_{x_{n}\in X_{n}}p\left(x_{1},\ldots ,x_{n}\right)log\left(p\left(x_{1},\ldots ,x_{n}\right)\right)$$

where $p(x_{1},\ldots ,x_{n})$ is the joint probability distribution of the variables. Since the joint entropy of a set of variables quantifies the total amount of the uncertainty or the information content of those variables, conditional entropy quantifies the information gained about a system of variables $X_{1},\ldots ,X_{n}$ when a subset of *k* variables $X_{i1},\ldots ,X_{ik}$ are known. Thus, the non-trivial problem of identifying the contribution of individual members to the overall structural variation becomes the task of computing the information overlap among their distance fields from an information theory point-of-view.

#### 4.2.2. Informative Isocontour Selection

As shown in Equation (9), we can use conditional entropy to identify the contribution of individual isocontours towards the overall uncertainty/entropy of the system of ensemble isocontours. Using a greedy approach, we iteratively select the contour which minimizes the uncertainty (i.e., entropy) left in the system. In each iteration, after a contour $X_{i}$ has been selected, the uncertainty left in the system is essentially the conditional entropy of the system of ensemble isocontours given that the selected contour is known, i.e., $H(X_{1},\ldots ,X_{n}|X_{i})$. The corresponding amount of entropy/uncertainty reduced by selecting a contour is referred to as its information gain in our work. Information gain of a member isocontour quantifies the informativeness of the member in the ensemble of isocontours. The sequence of isocontours, thus generated by the iterative process can be used to select a subset/sample of contours which can represent the uncertainty of the complete ensemble system.

**Algorithm 1** Informative Isocontour Selection Algorithm1:
$allVar:=[C^{0},C^{1},...,C^{n-1}]$

2:
infoVar:=emptystack
▹ list of informative contours3:
infoGain:=emptystack
▹ amount of information gain4:
**while**
allVar≠∅
**do**
▹ check the info gain of all contours5:    maxGain←−1.0
6:    importantVar←∅
7:    **for all** c in allVar
**do**
8:        infoVar.push(c)
9:        je←getJointEntropy(infoVar)▹ je: joint entropy10:        **if**
je>maxGain
**then**
11:           maxGain←je
12:           importantVar←c
13:        infoVar.pop()
14:    infoVar.push(importantVar)
15:    infoGain.push(maxGain)
16:    allVar.delete(maxVar)


Algorithm 1 explains the steps involved in constructing the sequence of informative isocontours. As shown in the pseudocode, the sequence of informative isocontours are pushed into the stack infoVar and the corresponding cumulative gain of information is pushed into another stack, infoGain. The procedure, getJointEntropy(infoVar) returns the joint entropy marginalized on the set of variables present in the parameter infoVar. getJointEntropy() queries from the joint histogram of the ensemble isocontours. Efficient construction of joint histograms of large number of variables is not a trivial task. A naive way to store joint histogram is to store it as a multi-dimensional array. However, as the number of variables increases, the memory cost of using multi-dimensional array increases exponentially, which makes this naive representation computationally prohibitive for a large number of variables. Lu et al. [41] presented a compact representation to store joint histogram by utilizing the sparse property. We use their approach to create a joint histogram for ensemble isocontours. Besides the storage benefits, this representation incorporates many histogram query operations as well. We only need to compute the joint histogram from all isocontours once, and then we can derive other histograms that are needed efficiently based on the histogram marginalization operation.

**Figure 4:**
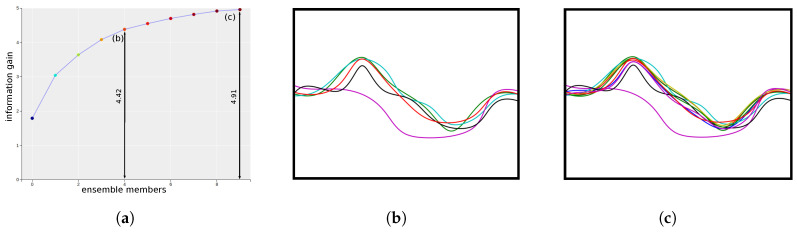
Informative isocontour selection in synthetic dataset: (**a**) Information gain curve for all 10 members. Each point on the plot corresponds to a member isocontour arranged from left to right in the descending order of their informativeness. The vertical axes show the maximum (cumulative) information gained about the system by selecting a sequence of members along the horizontal axis. (**b**) The isocontour plot of the Top 5 most informative isocontours. (**c**) The spaghetti plot of all 10 isocontours. As can be seen, the Top 5 isocontours (**b**) retain about 90% information of the complete system (**c**).

To facilitate such information guided exploration of the ensemble isocontours, we visualize the information gain values of selecting the ensemble members in a plot called the information gain curve (Figure 4a). The vertical axis of the plot represents the information gain while the horizontal axis represents the sequence of ensemble members. By following along the information gain curve from left to right, users can select the sequence of most informative ensemble members for a particular isovalue. Apart from visualizing the contribution of each members, this interactive information gain curve can also guide the users in selecting subsets of informative isocontours which can represent the total uncertainty of the system (including the anomalous members). In ensemble systems with hundreds of members, it is especially important to understand the most contributing members for quick analysis and simpler visualization.

Figure 4a shows the information gain curve for synthetically created 10 member isocontours. Bezier curves were drawn using varying control point locations to create this synthetic data. In this example, the total entropy/information of the system is 4.91. As can be seen, in the corresponding information gain curve, by selecting the first five informative members the information gained is 4.42 which is 90% information of the whole system. The spaghetti plots of the five most informative contours and all the 10 member contours are shown in Figure 4b,c, respectively. As can be seen, the five informative contour conveys almost the same uncertainty information as shown by all the members. This is particularly useful for systems with large number of members (which is shown in Section 5), as it helps in identifying the most important members relevant for uncertainty analysis for that isovalue.

## 5. Results

To demonstrate the effectiveness of our information-theoretic approach in exploring the scalar values and their ensemble isocontours, we tested it on three different types of ensemble datasets. The datasets have varying degrees of uncertainty and were selected from the field of material sciences, weather-forecasting and ocean-modeling. All the experiments were conducted on a standard workstation PC powered by Intel Core i7-2600 quad-core CPU running at 3.40 GHz with 16 GB of RAM.

### 5.1. Material Density Ensemble

Our first ensemble dataset was generated by performing multiple lock-exchange experiments [42] with different parameter setting. The experiment involves separating a light fluid from a heavy fluid with a barrier and then gradually letting them mix by releasing the barrier. We used a dataset with 100 ensemble simulation runs and a spatial resolution of 128×128.

**Figure 5:**
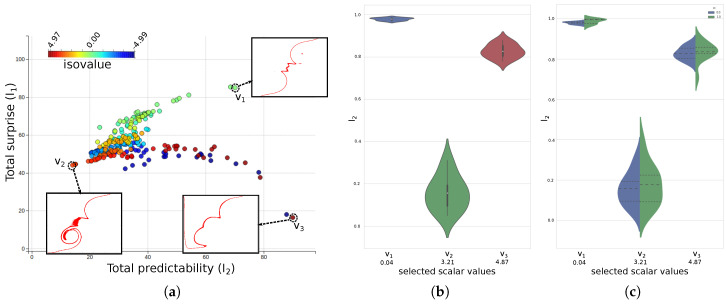
Scalar value exploration of material density ensemble: (**a**) Interactive Scatter-plot view of the total predictability vs. total surprise of the scalar values. $v_{1}$ corresponds to a scalar value with high predictability and high surprise; $v_{2}$ corresponds to low predictability and low surprisem i.e., high uncertainty; and $v_{3}$ corresponds to high predictability but low surprise. (**b**) Violin-plot view showing the distribution of individual predictability values for selected scalar values. (**c**) Split violin-plot view showing the distribution of the predictability values for the two directions of the bi-directional information channel.

Figure 5a shows the interactive scatter-plot view of the total predictability versus total surprise results for the scalar values, which are color-mapped to their values. Scalar values with high total predictability and high total surprise indicate that the corresponding ensemble isocontours of the scalar values are less uncertain, as is shown by the inset figure for the highlighted value $v_{1}$. On the other hand, for values with low predictability and surprise, the uncertainty of the ensemble isocontours are relatively high, as is shown for the highlighted value $v_{2}$. The third highlighted value, $v_{3}$, acts as an example for another interesting set of scalar values with high total predictability but low surprise. The high predictability of $v_{3}$ indicates that the corresponding ensemble isocontours are less uncertain, but the low surprise indicates high frequency of the value in the scalar fields, thus representing a relatively less important feature in the data. This is interesting because the set of values with high predictability and low surprise for this experiment corresponds to the boundary fluid-density values, i.e., the individual material densities of the two mixing fluids which dominates the scalar field. Regions with values close to these initial density values correspond to the locations where the materials have not yet mixed properly and is in fact not the feature that scientists are interested in studying in this experiment. Figure 5b,c shows the corresponding violin-plots for the user selected values in the scatter-plot. The *Y*-axis corresponds to the normalized predictability values of the individual members for the selected values which are plotted along the *X*-axis. The violin-plot conveys the contribution of individual member to the total predictability of a value. The shape of the violin glyph shows the distribution of the individual predictability values. The shape of the violins for values $v_{1}$ and $v_{3}$ in Figure 5b indicate a high agreement among the individual members, while the narrow violin for $v_{2}$ indicates the wide variation of the members. The second view of the violin-plot, as shown in Figure 5c, shows the distribution of individual predictability members for both the directions of the information channel. The blue side of the violin shows the distribution of how predictable are the individual members when the corresponding isocontour in the mean field is known, while the green side of the violin shows the distribution of predictability of the mean field when the corresponding isocontours in the individual members are known. A high variation in the distribution of the individual predictability values indicates that not all the isocontours contributed equally to the overall high uncertainty of that values. For such scalar values, we explore the importance of individual contours from an information-theoretic point-of-view.

**Figure 6:**
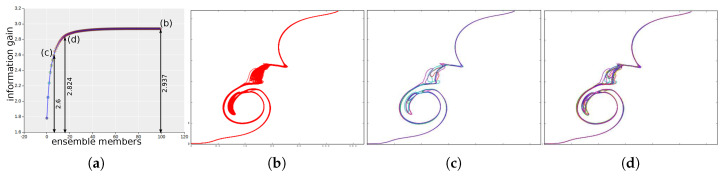
Informative isocontour exploration for material density value of 3.218: (**a**) The information gain curve for all 100 ensemble members. The vertical axes show the maximum information gained about the system by selecting a sequence of members in the plot. (**b**) The spaghetti plot of all 100 isocontours. (**c**) The isocontour plot of the Top 7 most informative isocontours. (**d**) The plot of Top 15 informative isocontours. Panels (**c**,**d**) are able to reveal the spatial layout of the isovalue with fewer members.

Figure 6a shows the information gain curve of the 100 member ensemble isocontours for isovalues 3.21 (i.e., $v_{2}$). The total entropy/uncertainty of the system is 2.9375. As can been seen, in the corresponding information gain curve, by selecting the first seven informative members, the information gained is 2.6, which is about 88.5% information about the system, while the information gained by selecting the first 15 informative members is 2.824 which is about 96% of the system. This says that the Top 15 most informative isocontours are sufficient to represent the structural variation of the 100 ensemble isocontours. We show this claim in subsequent figures by drawing the spaghetti plots of all the 100 isocontours (Figure 6b), Top 7 informative isocontours (Figure 6c) and the Top 15 informative isocontours (Figure 6d). As shown in Figure 6c,d, the spatial spread of the isocontours can be easily viewed with lesser number of informative isocontours. This offers a quicker way of understanding the uncertainty of the ensemble isocontours without looking at all the 100 instances. A supplementary video showing the interactive selection of scalar values from the scatter plot and the subsequent selection of informative ensemble isocontour members is provided along with this manuscript.

### 5.2. Great Lakes WRF Ensemble

Our second dataset was the Great Lakes WRF ensemble data, generated by the Atmospheric Sciences Program of the University of Wisconsin-Milwaukee. This is a nine-member ensemble of numerical weather forecasting across the Great Lakes region using the WRF-ARW forecasting model. The resolution of the dataset is 167 × 151. We used the pressure variable over the domain to perform our analysis.

**Figure 7:**
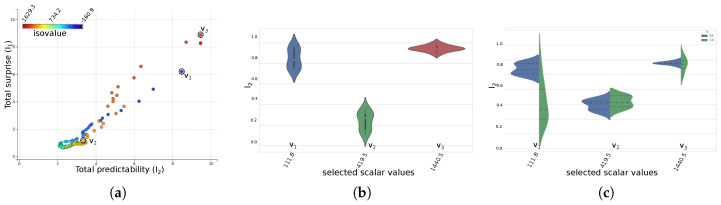
Scalar value exploration of Great Lakes WRF ensemble: (**a**) interactive scatter-plot view of the total predictability vs. total surprise of the scalar values; (**b**) violin-plot view showing the distribution of individual predictability values for selected scalar values; and (**c**) split violin-plot view showing the distribution of the predictability values for the two directions of the bi-directional information channel.

**Figure 8:**
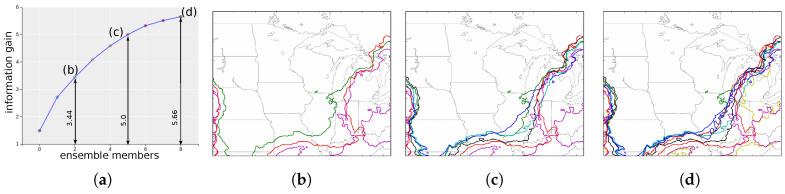
Informative isocontour selection of Great Lakes WRF ensemble: (**a**) the information gain curve for isovalue 1440.5; (**b**) the spaghetti-plot of the Top 3 informative isocontours which captures about 60% of the total uncertainty; (**c**) the spaghetti-plot of the Top 6 informative isocontours which captures about 88% of the total uncertainty; and (**d**) the spaghetti-plot of all the isocontours.

Figure 7a shows the total predictability versus surprise scatter-plot for the various pressure values. The values with high predictability and surprise corresponds to the less uncertain pressure values in the experiment. Figure 7b,c show the corresponding violin-plot views for the selected values in the scatter-plot, i.e., $v_{1}$ (111.8 Pa), $v_{2}$ (419.5 Pa) and $v_{3}$ (1440.5 Pa). These violin plots show the distribution of the predictability results of the individual simulation models for the selected pressure values. Figure 8a shows the information gain curve for isovalue 1440.5. As can be seen, the information gained about the ensemble isocontours is not significant by selecting just a few member. The total information/entropy of the system is 5.66 and the amount of information gained by selecting the Top 3 informative contours is 3.44 (about 60%) while selecting the Top 6 give an information gain of 5.0 (about 88%). Because of the high structural variations of the ensemble isocontours and relatively fewer ensemble members we cannot decide on a small subset of informative isocontours. As the spaghetti-plots of Top 3 (Figure 8b) and Top 6 (Figure 8c) show, the complete structural information of all nine ensemble isocontours (Figure 8d) cannot be represented properly by a subset of isocontours. This implies that for such datasets with high uncertainty and very low number of simulations, it is important to consider all the members for analysis.

### 5.3. Massachusetts Bay Ocean Modeling Ensemble

Our third dataset was a three-dimensional ensemble dataset, covering the region from the Massachusetts Bay to the Cape Cod area of the US east-coast [2,38,43]. This dataset is divided into 53×90 grid with 16 depth levels and consists of 25 ensemble simulations, generated by different sets of initial parameters and boundary conditions. The focus of this scientific experiment was to model the oceanic biodiversity of the selected region and to observe the effect of various variables on the life-forms. We used the oceanic temperature variable of the region to test our proposed technique for three-dimensional data.

**Figure 9:**
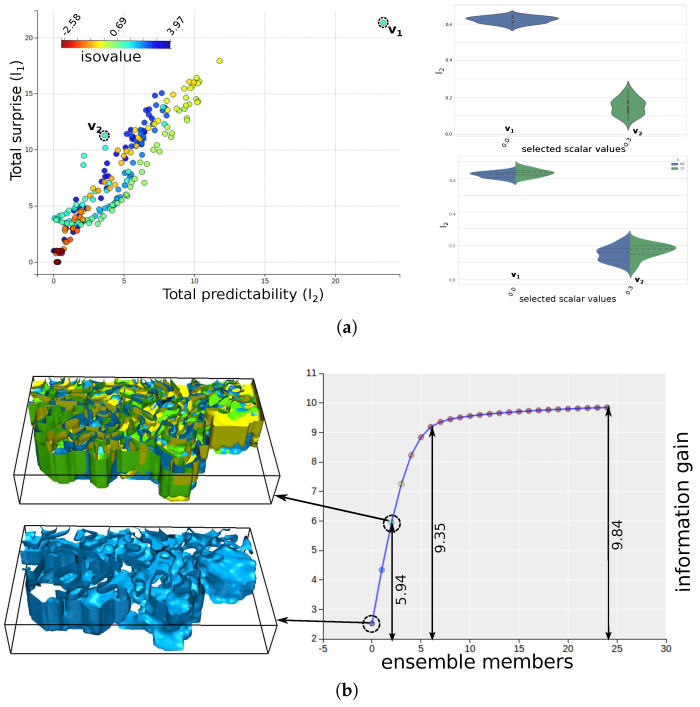
Information-theoretic exploration of ocean temperature values in the Massachusetts Bay ensemble dataset. (**a**) Interactive scatter-plot view of the total predictability vs. total surprise of the scalar values along with the violin-plot view for the selected values marked as $v_{1}$ and $v_{2}$. The violin-plot view (top right) shows the distribution of individual predictability values for $v_{1}$ and $v_{2}$. The split violin-plot view shows the distribution of the predictability values for the two directions of the bi-directional information channel; (**b**) The information gain curve for isovalue 0.0 for 25 members along with the ensemble isosurfaces. The bottom left isosurface corresponds to the most informative surface. The Top 3 informative isosurfaces are shown in top left image which comprises about 60% of the total uncertainty of all the members.

Figure 9a shows the total predictability versus total surprise plot for the various temperature values along with the two violin-plot views corresponding to the selected points in the scatter-plot. For this dataset, the structural variation of most of the values are very high. However, for the particular scalar value of 0.0${}^{\circ }$C, the relative predictability and surprise is high. This indicates a low uncertainty of its corresponding ensemble isosurfaces. Figure 9b shows the information gain curve for isovalue 0.0 alongside the ensemble isosurfaces selected in the curve. The curve shows the information gained about the system by selecting an ensemble isosurface member. We show the isosurface (blue) corresponding to the maximum information gain, i.e., the first point in the information gain curve. Since it is difficult to visualize multiple isosurfaces, we restrict to visualizing the Top 3 informative isosurfaces only: the blue surface is the most informative isosurface, the green is the second most informative isosurface and the yellow surface is the third most informative isosurface. These three isosurfaces represent about 60% of the information (structural variation) of the entire ensemble system. The inherent occlusion and clutter while drawing multiple isosurfaces make it a challenging problem to visually analyze multiple surfaces. Therefore, the kind of analysis and exploration that we have proposed in this work helps us understand the structural variation without going through the trouble of rendering multiple surfaces.

## 6. Discussion

**Figure 10:**
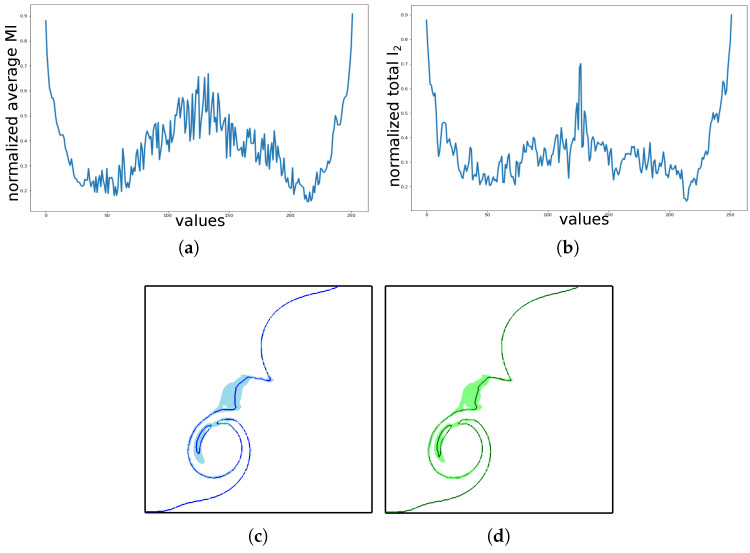
Validation: (**a**) The average pair-wise mutual information of ensemble isocontours for all the scalar values. (**b**) The total predictability results generated by our proposed method for all the scalar values. Both (**a**,**b**) reveal a similar trend of uncertainty across the value range. (**c**) Contour variability band of all 100 ensemble isocontours and (**d**) Top 7 informative isocontours. Both (**c**,**d**) display similar variability band structure and average contour shape.

Our proposed specific information based scalar value exploration method is an efficient and effective method to understand the variations of the ensemble isocontours of all the scalar values in the data. To the best of our knowledge, there is no other existing work that explores the isocontour uncertainty of a range of scalar values at the same time. A partially related work is the isosurface similarity map proposed by Bruckner et al. [16] that uses mutual information to compare the isosurfaces of all the scalar values in a single scalar field. Mutual information of the distance fields of the corresponding isosurfaces are computed for all the pairs of isovalues in the scalar field to create the similarity map. A similar approach for comparing the ensemble isocontours for all the scalar values will be exponentially expensive. However, to validate the predictability measures in our work, we compared our results with a similar brute force mutual information based approach for the 100 member material density dataset. We extracted the individual isocontours and their distance fields for all the corresponding scalar values and computed the pair-wise mutual information values across all the 100 members. Figure 10a shows the normalized average pair-wise mutual information values for all the scalar values, while Figure 10b shows the normalized total predictability ($I_{2}$) computed by our proposed method. We can see similar uncertainty trend for the range of scalar values. However, the brute force approach took about 49 minutes and our specific information based approach took about 1.4 minutes for all the scalar values across 100 ensemble members.

For a single scalar value, there are many ensemble isocontour visualization techniques [4,5,6,7]. All these techniques can be used to understand the uncertainty associated with a selected isovalue. The first stage of our exploration helps the user to select scalar values for such analysis. However, current analysis methods do not offer any insights into how the individual member isocontours of a scalar value are contributing to the overall uncertainty. This is addressed in the second stage of our exploration, where we use conditional entropy to determine the informativeness of the individual member isocontours. To the best of our knowledge, there is no existing work in ensemble visualization literature that quantifies the uncertainty contribution of individual members. The informativeness results help us create samples/subsets of members that can reliably represent the uncertainty of the complete set of ensemble isocontours. This is especially useful for systems with large number of members. Apart from the visual validations shown via the spaghetti-plots in Section 5, we tried to validate whether the subsets we chose were indeed representative of the original ensemble. To do so, we visualized the distribution of isocontours by drawing bands using the algorithm proposed by Ferstl et al. [6]. Figure 10c shows the band and the geometric median created by the original 100 ensemble isocontours of the material density dataset, while, Figure 10d shows the band and the geometric median created by the Top 7 informative isocontours selected by our algorithm. The band of Top 7 contours covers 91% of the original band-area. This shows that our approach selects samples which are good representation of the structural properties of the original ensemble.

In Table 1, we show the computation times of the major steps. The second column of the table shows the data resolution and corresponding number of ensemble member in parenthesis. The third column shows the computational time for the calculation of the $I_{1}$ and $I_{2}$ values across all the information channels. The fourth column shows the time for conditional entropy based informative isocontour computation for a single scalar value.

Although these computations are one-time activities for a given ensemble dataset, we feel that there is room for improvement with respect to performance. Parallelization of the entire pipeline can help us in achieving better performances.

## 7. Conclusions and Future Work

In this paper, we present an information-theoretic approach of exploring the uncertainties across a scalar value range for ensemble datasets. Further, for a single scalar value, we let the users explore the structural variations of the ensemble isocontours. Using specific information measures, $I_{1}$ and $I_{2}$, we propose a method to understand the effect of uncertainty on the ensemble isocontours of all the scalar values. For a selected scalar value, we let the users explore the structural variations of the ensemble isocontours using a conditional entropy based method. This exploration leads to the identification of structurally informative isocontours that can represent the spatial properties of the complete ensemble system.

In the future, we plan to extend this method to time-varying and multivariate ensemble datasets. The current method is targeted only for ensemble scalar datasets, however similar specific information measures can also be applied for vector datasets to understand the uncertainty of various features such as streamlines and stream-surfaces.

## Figures and Tables

**Table 1:** Performance of various computational stages.

Datasets	Dim (Ensembles)	$I_{1}$ and $I_{2}$ (secs)	Cond. Entropy (secs)
Material Density	128×128(100)	84.3	47.3
Great Lake	167×151(9)	1.2	20.5
Mass. Bay	16×53×90(25)	5.1	33.2

## Supplementary Materials

The Supplementary Materials are available online at http://www.mdpi.com/1099-4300/20/7/540/s1. Supplementary Video S1.

## References

[1] Bonneau G.P., Hege H.C., Johnson C.R., Oliveira M.M., Potter K., Rheingans P., Schultz T., Hansen D.C., Chen M., Johnson R.C., Kaufman E.A., Hagen H. (2014). Overview and State-of-the-Art of Uncertainty Visualization. Scientific Visualization: Uncertainty, Multifield, Biomedical, and Scalable Visualization.

[2] Love A.L., Pang A., Kao D.L. (2005). Visualizing spatial multivalue data. IEEE Comput. Graph. Appl..

[3] Pang A., Wittenbrink C., Lodha S. (1996). Approaches to Uncertainty Visualization.

[4] Whitaker R.T., Mirzargar M., Kirby R.M. (2013). Contour boxplots: A method for characterizing uncertainty in feature sets from simulation ensembles. IEEE Trans. Vis. Comput. Graph..

[5] Sanyal J., Zhang S., Dyer J., Mercer A., Amburn P., Moorhead R.J. (2010). Noodles: A tool for visualization of numerical weather model ensemble uncertainty. IEEE Trans. Vis. Comput. Graph..

[6] Ferstl F., Kanzler M., Rautenhaus M., Westermann R. (2016). Visual Analysis of Spatial Variability and Global Correlations in Ensembles of Iso-Contours. Comput. Graph. Forum (Proc. Eur. Vis.).

[7] Pöthkow K., Weber B., Hege H.C. Probabilistic Marching Cubes. Proceedings of the 13th Eurographics/IEEE-VGTC Conference on Visualization.

[8] Hintze J.L., Nelson R.D. (1998). Violin Plots: A Box Plot-Density Trace Synergism. Am. Stat..

[9] Cover T.M., Thomas J.A. (2006). Elements of Information Theory; Wiley Series in Telecommunications and Signal Processing.

[10] Chen M., Sbert M., Shen H.W., Viola I., Bardera A., Feixas M., Sousa A., Bouatouch K. (2016). Information Theory in Visualization.

[11] Wang C., Yu H., Ma K. (2008). Importance-Driven Time-Varying Data Visualization. IEEE Trans. Vis. Comput. Graph..

[12] Chen M., Jänicke H. (2010). An Information-theoretic Framework for Visualization. IEEE Trans. Vis. Comput. Graph..

[13] Xu L., Lee T.Y., Shen H.W. (2010). An Information-Theoretic Framework for Flow Visualization. IEEE Trans. Vis. Comput. Graph..

[14] Pluim J.P.W., Maintz J.B.A., Viergever M.A. (2003). Mutual-information-based registration of medical images: A survey. IEEE Trans. Med. Imaging.

[15] Huang X., Paragios N., Metaxas D.N. (2006). Shape Registration in Implicit Spaces Using Information Theory and Free Form Deformations. IEEE Trans. Pattern Anal. Mach. Intell..

[16] Bruckner S., Moller T. Isosurface Similarity Maps. Proceedings of the 12th Eurographics/IEEE-VGTC Conference on Visualization, EuroVis’10.

[17] Wei T.H., Lee T.Y., Shen H.W. Evaluating Isosurfaces with Level-set-based Information Maps. Proceedings of the 15th Eurographics Conference on Visualization EuroVis ’13.

[18] Deweese M.R., Meister M. (1999). How to measure the information gained from one symbol. Netw. Comput. Neural Syst..

[19] Bramon R., Boada I., Bardera A., Rodriguez J., Feixas M., Puig J., Sbert M. (2012). Multimodal Data Fusion Based on Mutual Information. IEEE Trans. Vis. Comput. Graph..

[20] Dutta S., Liu X., Biswas A., Shen H.W., Chen J.P. Pointwise Information Guided Visual Analysis of Time-varying Multi-fields. Proceedings of the SIGGRAPH ASIA 2017 Symposium on Visualization.

[21] Biswas A., Dutta S., Shen H.W., Woodring J. (2013). An Information-Aware Framework for Exploring Multivariate Data Sets. IEEE Trans. Vis. Comput. Graph..

[22] Brown G., Pocock A., Zhao M.J., Luján M. (2012). Conditional Likelihood Maximisation: A Unifying Framework for Information Theoretic Feature Selection. J. Mach. Learn. Res..

[23] Nguyen X.V., Chan J., Romano S., Bailey J. (2014). Effective Global Approaches for Mutual Information Based Feature Selection. Proceedings of the 20th ACM SIGKDD International Conference on Knowledge Discovery and Data Mining.

[24] Johnson C.R., Sanderson A.R. (2003). A Next Step: Visualizing Errors and Uncertainty. IEEE Comput. Graph. Appl..

[25] Djurcilov S., Kim K., Lermusiaux P., Pang A. (2002). Visualizing Scalar Volumetric Data with Uncertainty. Comput. Graph..

[26] Potter K., Rosen P., Johnson C.R. (2012). From quantification to visualization: A taxonomy of uncertainty visualization approaches. Uncertainty Quantification in Scientific Computing.

[27] Molteni F., Buizza R., Palmer T., Petroliagis T. (1996). The ECMWF Ensemble Prediction System: Methodology and validation. Q. J. R. Meteorol. Soc..

[28] Miyoshi T., Kondo K., Terasaki K. (2015). Big Ensemble Data Assimilation in Numerical Weather Prediction. Computer.

[29] Obermaier H., Joy K. (2014). Future challenges for ensemble visualization. Comput. Graph. Appl. IEEE.

[30] Wang J., Hazarika S., Li C., Shen H.W. (2018). Visualization and Visual Analysis of Ensemble Data: A Survey. IEEE Trans. Vis. Comput. Graph..

[31] Potter K., Wilson A., Bremer P.-T., Williams D., Doutriaux C., Pascucci V., Johhson C. (2009). Visualization of uncertainty and ensemble data: Exploration of climate modeling and weather forecast data with integrated ViSUS-CDAT systems. J. Phys. Conf. Ser..

[32] Demir I., Dick C., Westermann R. (2014). Multi-Charts for Comparative 3D Ensemble Visualization. IEEE Trans. Vis. Comput. Graph..

[33] Alabi O.S., Wu X., Harter J.M., Phadke M., Pinto L., Petersen H., Bass S., Keifer M., Zhong S., Healey C., Taylor R.M. (2012). Comparative Visualization of Ensembles Using Ensemble Surface Slicing.

[34] Hazarika S., Dutta S., Shen H.W. Visualizing the variations of ensemble of isosurfaces. Proceedings of the 2016 IEEE Pacific Visualization Symposium (PacificVis).

[35] Pöthkow K., Hege H.C. (2011). Positional Uncertainty of Isocontours: Condition Analysis and Probabilistic Measures. IEEE Trans. Vis. Comput. Graph..

[36] Pöthkow K., Petz C., Hege H.C. (2013). Approximate level-crossing probabilities for interactive visualization of uncertain isocontours. Int. J. Uncertain. Quantif..

[37] Hazarika S., Biswas A., Shen H.W. (2018). Uncertainty Visualization Using Copula-Based Analysis in Mixed Distribution Models. IEEE Trans. Vis. Comput. Graph..

[38] Lermusiaux P.F., Chiu C.S., Gawarkiewicz G.G., Abbot P., Robinson A.R., Miller R.N., Haley P.J., Leslie W.G., Majumdar S.J., Pang A. (2006). Quantifying Uncertainties in Ocean Predictions.

[39] Kullback S., Leibler R.A. (1951). On information and sufficiency. Ann. Math. Stat..

[40] Jones M.W., Baerentzen J.A., Sramek M. (2006). 3D Distance Fields: A Survey of Techniques and Applications. IEEE Trans. Vis. Comput. Graph..

[41] Lu K., Shen H.W. A compact multivariate histogram representation for query-driven visualization. Proceedings of the 2015 IEEE 5th Symposium on Large Data Analysis and Visualization (LDAV).

[42] Micard D., Dossmann Y., Gostiaux L. Mixing Efficiency in a Lock Exchange Experiment. Proceedings of the International Symposium on Stratified Flows.

[43] Lermusiaux P.F. (2006). Uncertainty estimation and prediction for interdisciplinary ocean dynamics. J. Comput. Phys..

